# Supplementary material for: Saccade-synchronized rapid attention shifts in macaque visual cortical area MT
Source: Nat Commun. 2018 Mar 6;9:958. doi: 10.1038/s41467-018-03398-3 (PMC5840291; doi:10.1038/s41467-018-03398-3)
Supplement: Supplementary file 1 — Supplementary Information [file 41467_2018_3398_MOESM1_ESM.pdf]

# Supplementary Information

## Yao et al. : Saccade-synchronized rapid attention shifts in macaque visual cortical area MT

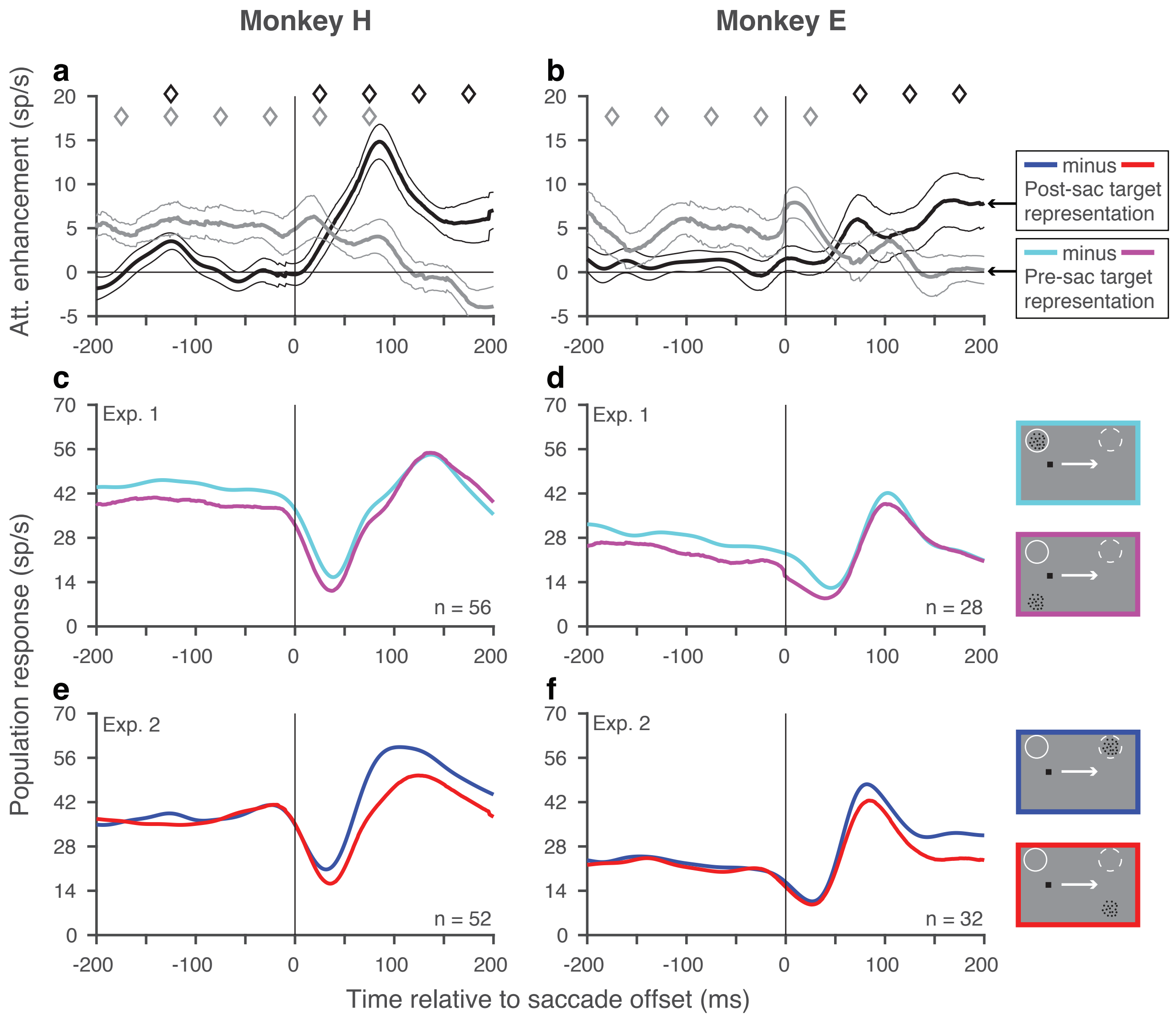


**Supplementary Figure 1. The attention shift remains saccade-synchronized when the average firing-rates are matched between the populations in Experiment 1 and 2. Related to Figure 2**. Figure format identical to Figure 2, except that neurons are dropped from the analysis in order to match the average firing-rate distributions in Experiment 1 and 2 for each 5 Hz bin (starting at 0 Hz). As in Figure 2, the panels show mean and s.e.m. of firing-rate differences (a-b) and mean firing-rates (c-f). The average firing-rate was calculated between -600 and 600 ms after saccade offset and averged over both attend-in and attend-out conditions. The mean-matching was done separately for each monkey: 33 neurons (monkey H) and 16 neurons (monkey E) were retained following the mean-matching procedure. The attentional cross-over times now occur at 35 ms and 50 ms after saccade offset, compared to 29 and 53 ms in the full sample in Figure 2.

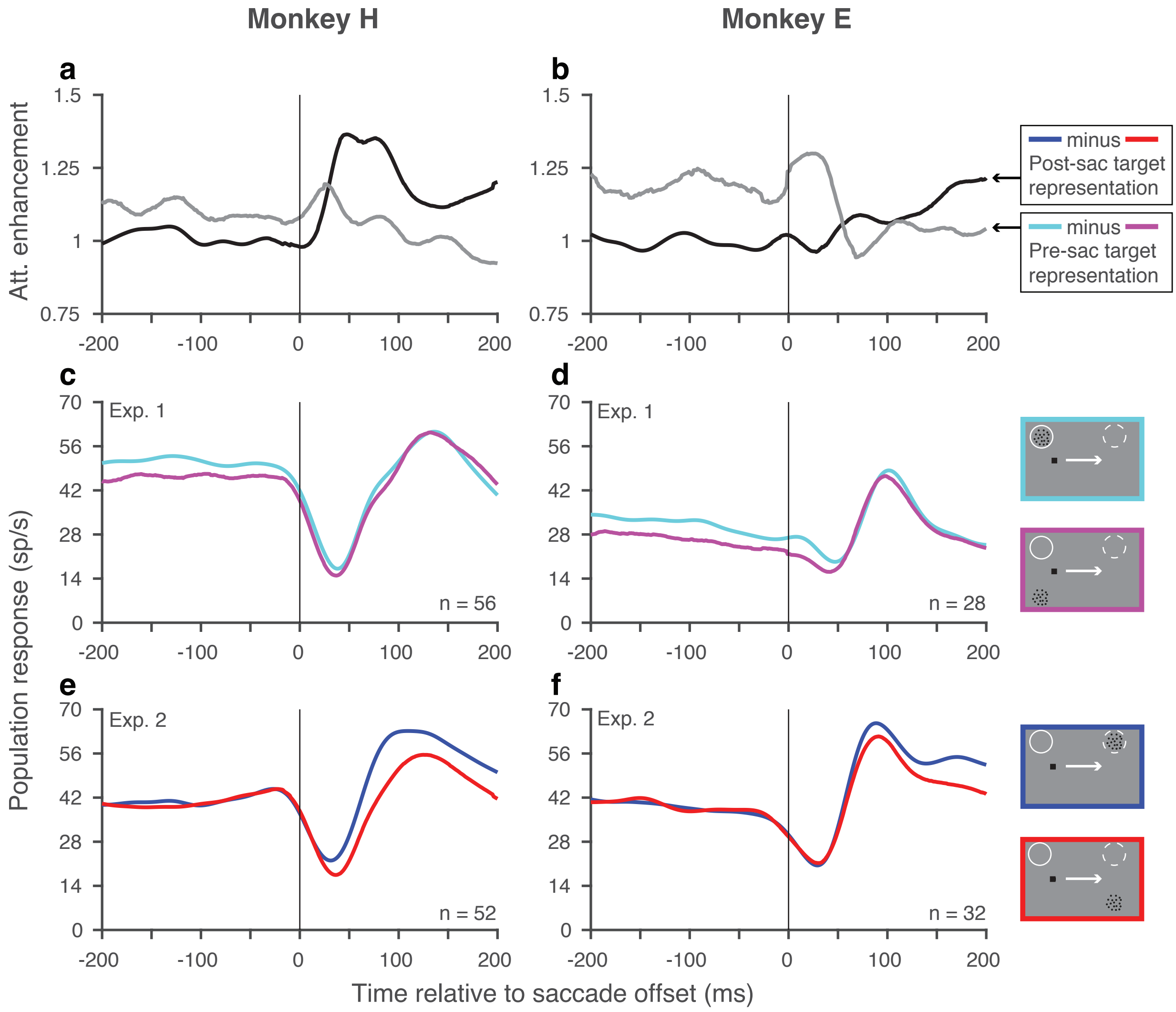


**Supplementary Figure 2. The attention shift remains saccade-synchronized when the attentional enhancement is estimated as a ratio (based on a multiplicative attentional effect), rather than a difference in firing-rates. Related to Figure 2.** Figure identical to Figure 2, except that the ratio of the curves in c-f is plotted in a-b. The panels show the ratio of mean firing-rates (a-b) and mean firing-rates (c-f). The attentional cross-over times now occur at 30 and 55 ms after saccade offset, compared to 29 and 53 ms after saccade offset in Figure 2. Computing the ratio between the average PSTHs, though slightly biased, is a reasonable estimator of the true population ratio and in our simulations, performs as well as the different corrected measures that have been proposed (Rao and Rao, 1971), and performs much better than an estimator based on the conventional attentional index.

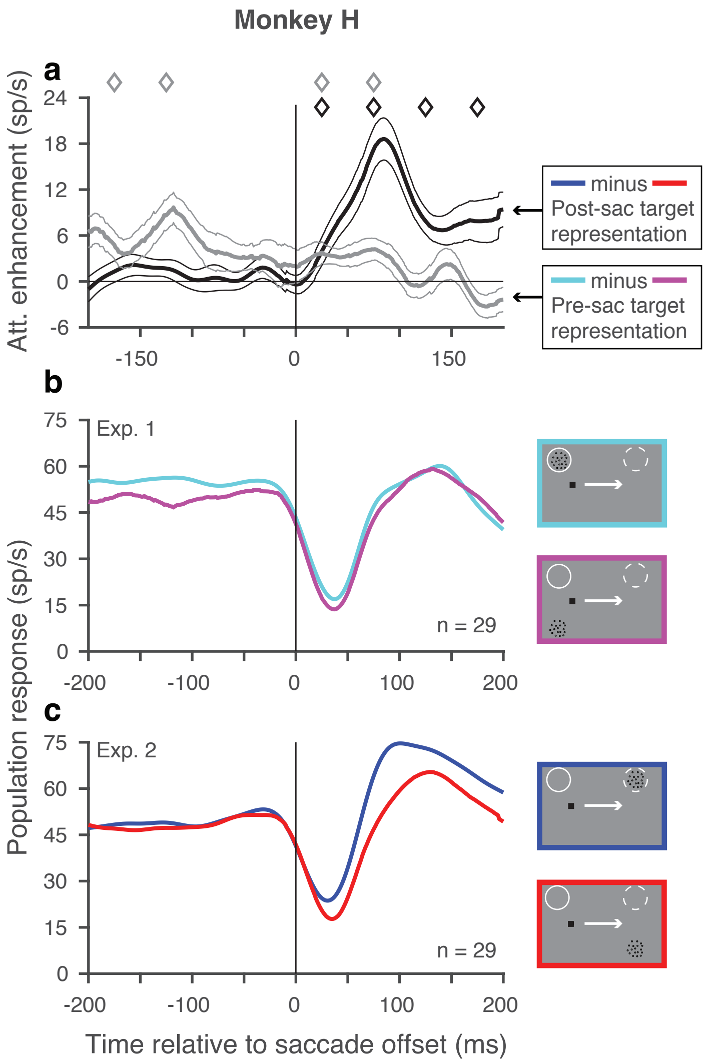


**Supplementary Figure 3. The attention shift remains saccade-synchronized when only the neurons recorded from both Experiments 1 and 2 are analyzed. Related to Figure 2.** Figure format identical to Figure 2, except that only the 29 neurons with data from both Experiments 1 and 2 are considered. All these neurons are from monkey H. As in Figure 2, the panels show mean and s.e.m. of firing-rate differences (a) and mean firing-rates (b-c). The attentional cross-over times now occurs at 24 ms after saccade offset, compared to 29 ms for the full sample from monkey H in Figure 2a.

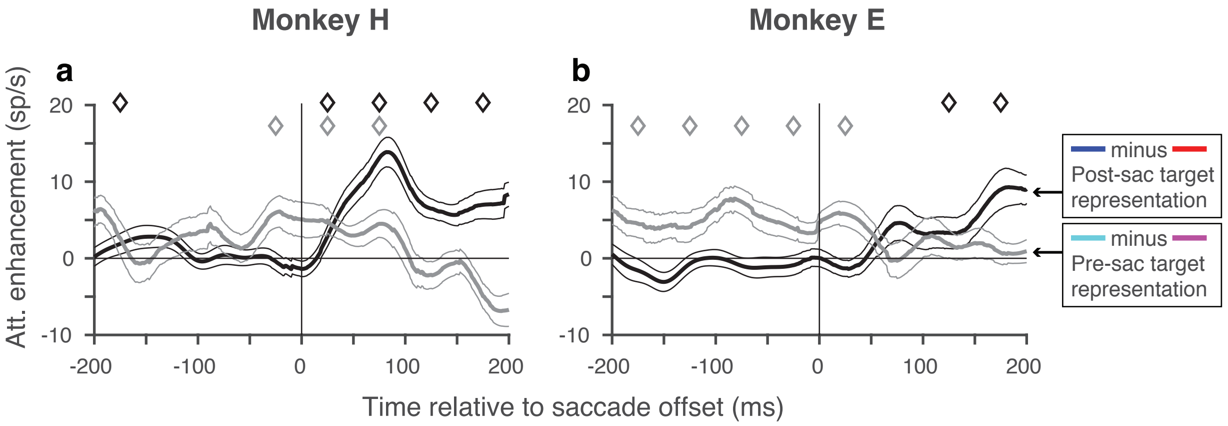


**Supplementary Figure 4. The post-saccadic time-course of attention shifts remains similar when latency distributions were matched (by dropping trials) between the attend-in and attend-out conditions. Related to Figure 2.** Figure format identical to Figure 2 a-b, except that the reduced dataset (after dropping trials for latency matching) of Figure 3 was used. As in Figure 2 a-b, the panels show mean and s.e.m. of firing-rate differences (a-b). The attentional cross-over times (after saccade offset) now occur at 31 ms and 57 ms after saccade offset, compared to 29 ms and 53 ms for the full sample in Figure 2.

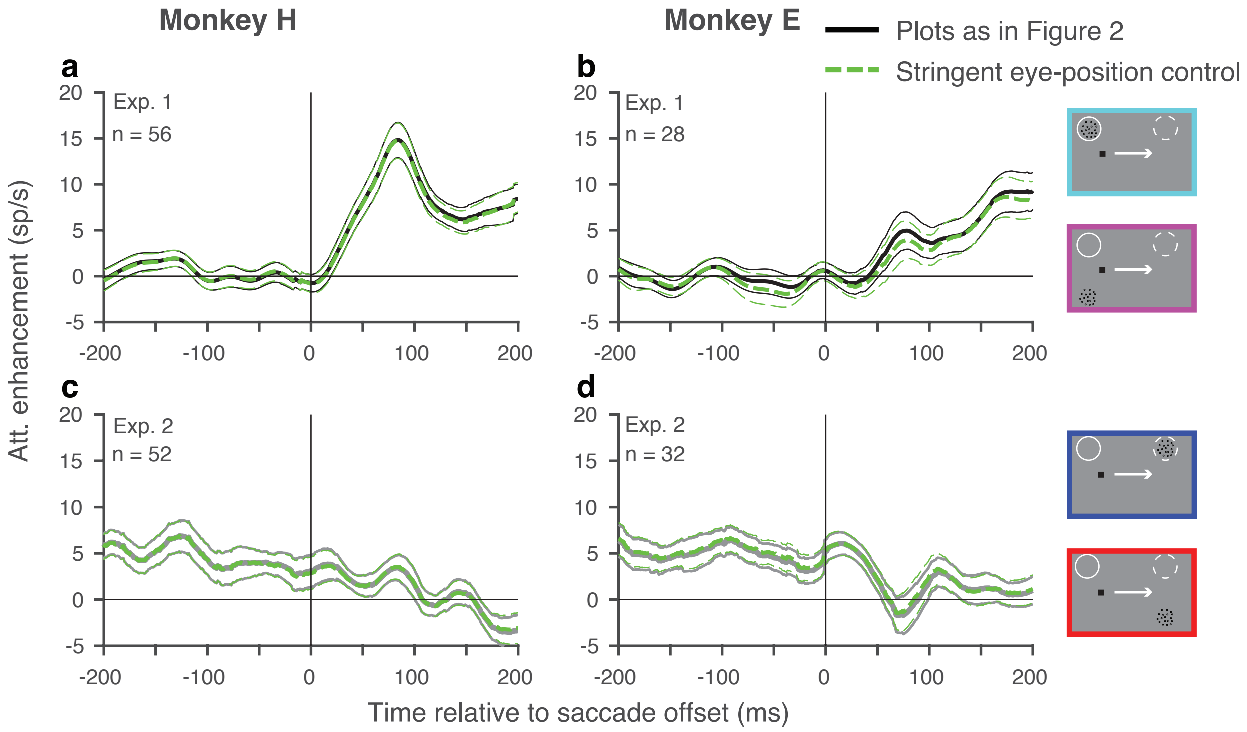


**Supplementary Figure 5. The time-course of the attention shifts is minimally affected when the saccadic behavior is constrained more stringently. Related to Figure 2.** Figure format identical to Figure 2 a-b, except that the grey curves in Fig. 2 a and b are now plotted in c and d respectively. Also, superimposed on all 4 plots (a-d) are green curves, which are calculating using only those trials where the saccade ended within 2 degrees of the post-saccadic fixation position, and no additional saccades were made between 0 and 100 ms following saccade offset (the time during which the attentional crossing point occurs). 97 % and 90.6 % of trials were included with the more stringent criterion (in monkey H and E respectively). Error bars show s.e.m.

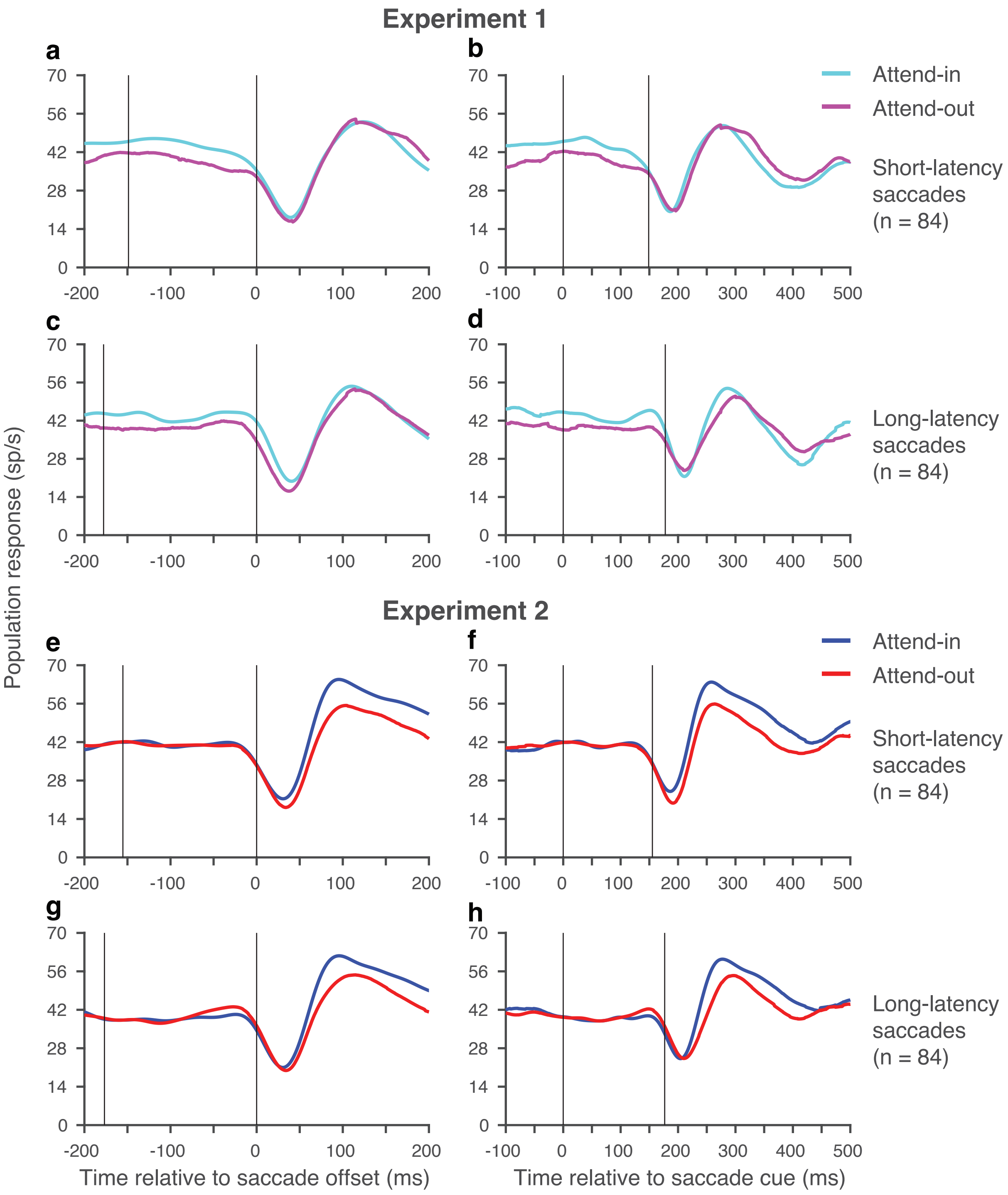


**Supplementary Figure 6. The time-course of attention shift is similar when aligned to saccade offset, not when aligned to fixation point offset. Related to Figure 3.** PSTHs from which the difference curves in Figure 3a-d were derived. Data plotted for trials with saccade latencies shorter than the median (a-b, e-f) and longer than the median (c-d, g-h) for the corresponding task conditions in Experiment 1 (a-d) and Experiment 2 (e-h). Left column shows data aligned to saccade offset and the right column shows data aligned to fixation point offset. Vertical lines away from 0 indicate the mean time of fixation point offset (left column) and the mean saccade latency (right column). As in Figure 3, data from both monkeys were pooled for this analysis and the same dataset (after matching for saccade offset timing) was used.

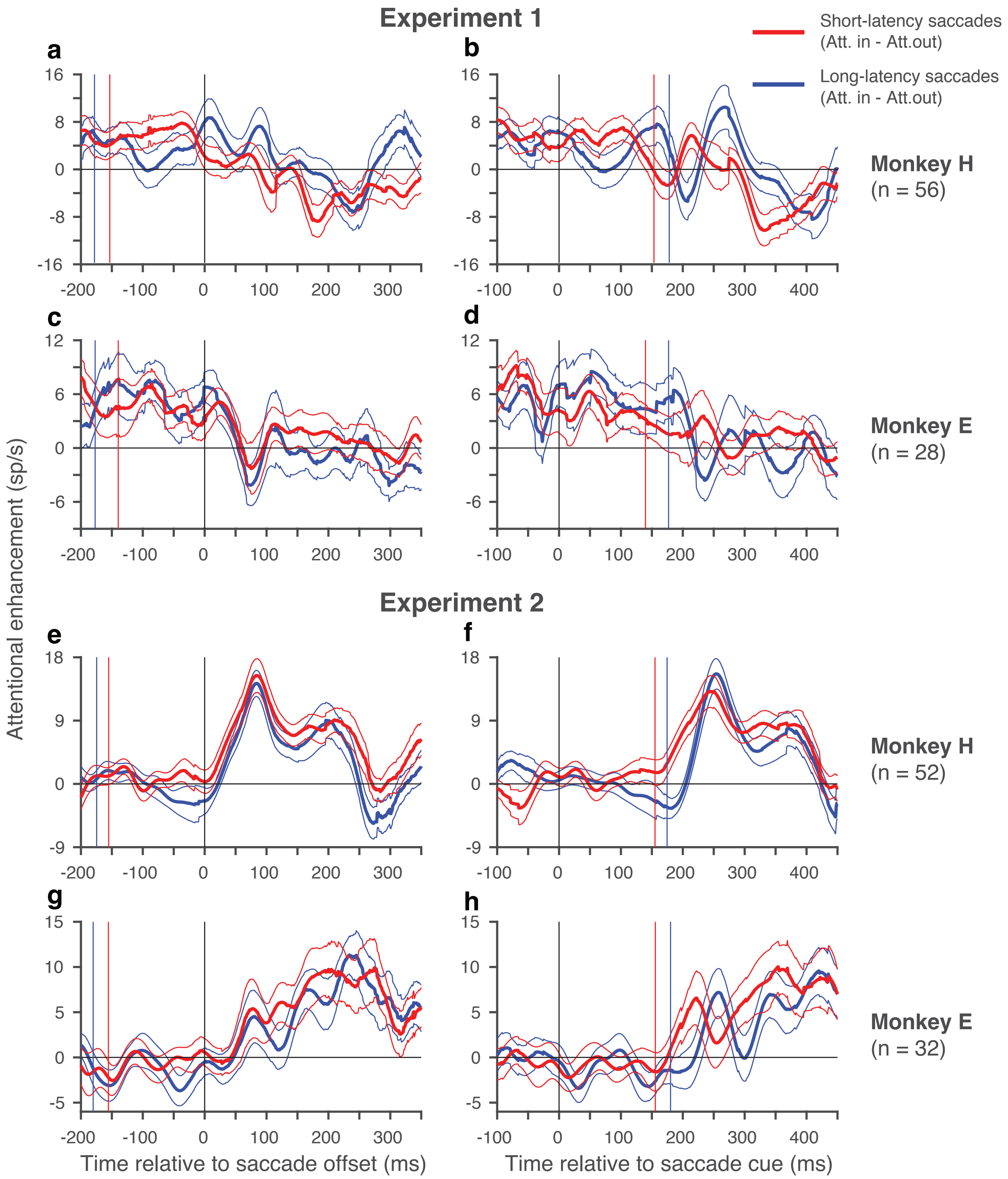


**Supplementary Figure 7. The patterns in the pooled data of Figure 3 are generally consistent with those in the individual monkeys. Related to Figure 3.** Figure format identical to Figure 3, except that instead of the pooled data in Figure 3, data are shown separately for monkey H (a-b,e-f) and monkey E (c-d,g-h). The attention shift's time-course remained superimposed when aligned to saccade offset for both monkeys (a,c,e and g). When aligned to fixation point offset, the time-course of attentional enhancement in both monkeys showed the same delay pattern for long-latency saccade trials seen in the pooled data (b,d,f and h), as expected if the attention shift was co-ordinated with saccade planning/execution. Y-axis limits are the same for each row, but are adjusted across rows to allow the delays in the noisy patterns to be visualized more easily. The same dataset (after matching for saccade offset timing) was used as in Figure 3. Error bars show s.e.m.

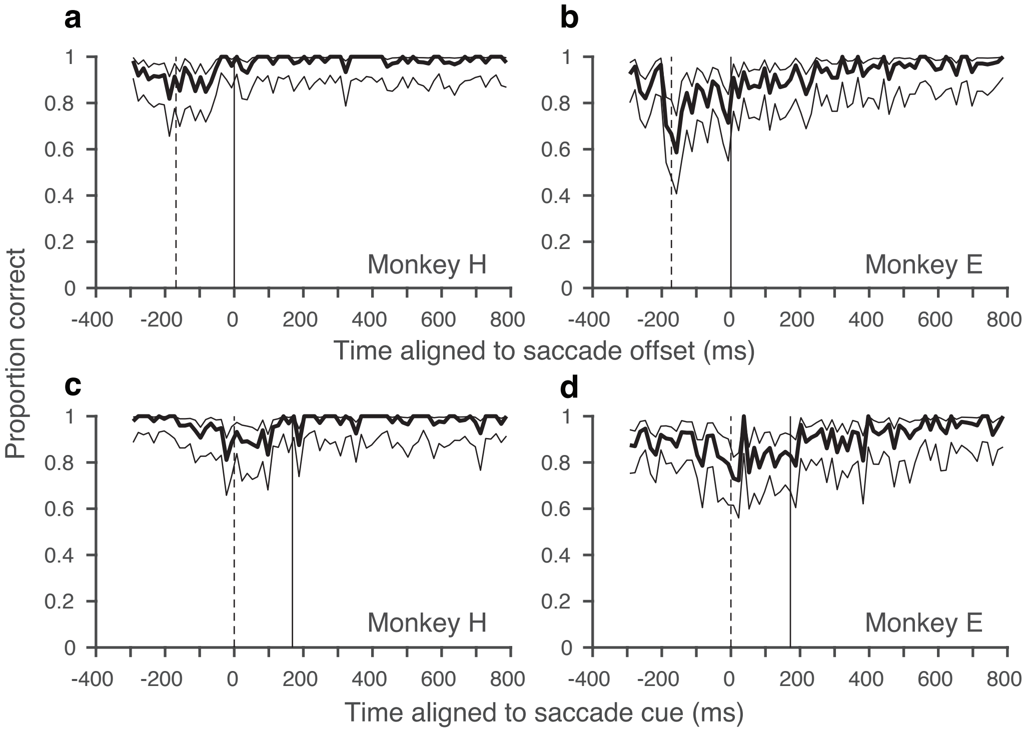


**Supplementary Figure 8. The target-change detection performance shows minimal peri-saccadic modulation.** The target-detection performance for monkey H (a,c) and monkey E (b,d) is plotted aligned to saccade offset (a-b) and to the saccade cue onset (i.e. time of fixation point jump; c-d). Performance is computed in non-overlapping 15 ms bins and plotted along with Wilson score-intervals for the proportion. The solid vertical line shows either the time of saccade offset (at 0 in a-b) or the mean time of saccade offset (c-d); the dotted vertical line shows the time of saccade cue onset (at 0 in c-d) or the mean time of saccade cue onset (a-b). The performance showed minimal variation around saccade offset (a-b), and specifically in the critical period from 0 to 100 ms following saccade offset when the attentional cross-over takes place. The performance is also plotted relative to the saccade cue onset time for comparison (c-d); compared to a-b, this plot includes some additional trials (with target-change times in the early portion of the displayed curve) where the monkeys responded without executing a saccade. Performance plotted from 9064 trials in 47 sessions (monkey H) and 10386 trials in 40 sessions (monkey E).

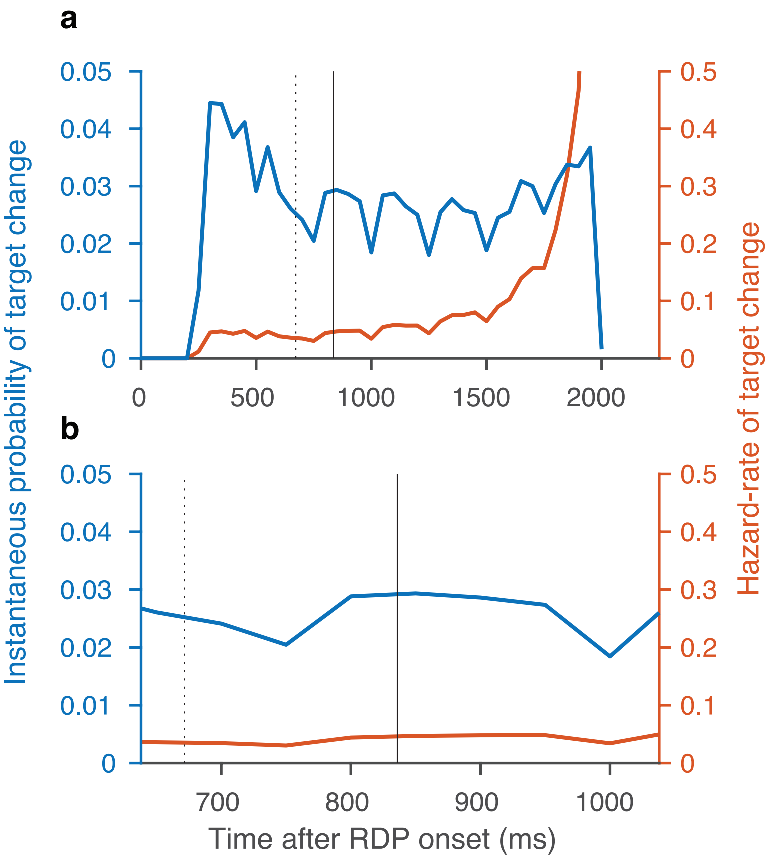


**Supplementary Figure 9. The hazard function for the response event (the target-change) changes minimally around saccade offset.** The time-course of attentional shift is unlikely to be related to changes in the monkeys' expectancy of the target-change, since the hazard function for target change does not vary markedly during the critical period (-200 to 200 ms around saccade offset). The distribution of target-change times was identical for Experiments 1 and 2 and is plotted in blue (a,b). The corresponding hazard-rate is plotted in red on the same axes. The plots in b are identical to those in a, but with the X-axis restricted to the 200 ms period on either side of the mean time of saccade offset (black vertical line). The dotted vertical line shows the mean time of saccade cue onset.

## Supplementary Note 1

*Saccade latency*

There were often differences in saccadic latency between the attention conditions, due to interactions between the RF location and the saccade target location with respect to the fixation point. These differences were however usually small and unsystematic: for Experiments 1 and 2 in monkey E, and Experiment 2 in monkey H, 90 % of the sessions had mean latency differences (between the two attentional conditions) that lay below 12.5 ms. The mean differences across sessions were also small and not significant: Experiment 2 in monkey H, 1.3 ms shorter (SEM=0.7 ms), Experiment 1 in monkey E, 5.5 ms shorter (SEM=3.7 ms) and Experiment 2 in monkey E, 4.5 ms shorter (SEM=2.3 ms). The only exception was for Experiment 1 in monkey H. Here, the cutoff value was larger: 90 % of the sessions had mean latency differences below 33.5 ms. Also, these differences were clearly systematic: the saccade latency in the attend-in condition was 20.5 ms shorter than that for the attend-out condition (SEM=1.4 ms).

There are two reasons why these latency differences are unlikely to influence our conclusions. First, they were generally small and non-significant, and even in the case of Experiment 1 in Monkey H, the differences were still of the order of 20-30 ms. Second, and more importantly, the attention-shifts in the data are saccade-synchronized, and therefore these latency differences in the timing of the saccade do not affect the data when viewed aligned to saccade-offset (though they do affect the interpretation when aligned to saccade cue-onset). In any case, we redid the analysis in Figure 2, using the reduced mean-matched dataset from Figure 3, where for each session, trials were dropped from each condition in order to match the distribution of latencies (within 20 ms bins). Though only 66 % of trials were now retained (overall), the basic features of the attentional time-course in the 0-100 ms period after saccade offset remain robust (Supplementary Figure 4).

*Saccade accuracy*

We first calculated a final eye-fixation position by taking the median eye-position from 125 ms after the saccade end to the target change time across trials as the final eye-fixation position (this corrected for small eye-calibration errors). 98.9 and 98.2 % of saccades ended within 3 degrees of this final eye-position in monkeys H and E respectively; the values for a 2 degree range were 97.1 % and 89.7 %, and for a one-degree range were 72.6 % and 52.6 %. The saccades in monkey H thus ended closer to the final fixation position than in monkey E. Both monkeys showed additional changes in eye-position (drifts and small saccades) after acquiring the saccade target: this is not surprising given that they were performing a dual task (fixate and attend to the peripheral target RDP to detect a small change). Consistent with the better saccade accuracy in monkey H compared to monkey E, the first corrective saccade (of larger than 1 degree amplitude to avoid small microsaccades and dynamic overshoots) in monkey H ended only 0.04 degrees closer on average to the final fixation position (SEM=0.016 degrees) compared to its starting position. In monkey E, this value was 0.8 degrees (SEM=0.046 degrees). A similar result was obtained when calculating over all saccades during the post-saccadic fixation period: these saccades ended 0.19 degrees (SEM=0.01 degrees) closer in monkey H and 0.81 degrees (SEM=0.04 degrees) closer in monkey E. We also note that the accuracy was also quite consistent across attention conditions and thus accuracy differences do not impact our results: the mean difference between the saccade accuracy in the attend-in and attend-out conditions was very small (Experiment 1, monkey H: 0.07 degrees (SEM=0.04 degrees), monkey E: -0.005 degrees (SEM=0.03 degrees); Experiment 2, monkey H: 0.07 degrees (SEM=0.03 degrees), monkey E: -0.13 degrees (SEM=0.05 degrees).

## **Supplementary References**

(see Supplementary Figure 2 legend)

1. Rao PSRS, Rao JNK. Small sample results for ratio estimators. Biometrika, 58, 625-30, 1971.
